# Characterizing Interventions Used to Promote Life Participation in Adults on Peritoneal Dialysis Therapy: A Scoping Review

**DOI:** 10.1177/20543581241263168

**Published:** 2024-07-30

**Authors:** Alexia Kateb, Kaleigh McCarthy, Janine Farragher

**Affiliations:** 1 Department of Occupational Science and Occupational Therapy, University of Toronto, ON, Canada

**Keywords:** kidney failure, peritoneal dialysis, life participation, rehabilitation, quality of life

## Abstract

**Background:**

Living with kidney failure can interfere with life participation (ie, participation in valued life activities). Life participation has recently been identified as a top-priority health outcome of people on peritoneal dialysis therapy, but it is a relatively unexplored topic in peritoneal dialysis.

**Objective:**

The objective is to describe the interventions that have been used to promote life participation in the peritoneal dialysis population and highlight research gaps warranting further investigation.

**Design:**

A scoping review was conducted according to the Joanna Briggs Institute methodology.

**Setting:**

Six electronic databases (MEDLINE [OVID], EMBASE, PsycINFO, Cochrane Central Register of Controlled Trials, CINAHL Plus, SCOPUS) were searched.

**Patients:**

Adults aged 18+ years on peritoneal dialysis therapy.

**Measurements:**

Any dedicated scale or subscale that measured life participation as an isolated outcome.

**Methods:**

Title/abstract screening was completed independently after adequate inter-rater reliability (kappa > 0.8) was achieved among reviewers. Full-text review and data extraction were conducted in duplicate. Extracted data were analyzed using counts, percentages, and narrative synthesis to describe patterns in the literature.

**Results:**

After identifying 13 874 results, 17 studies met eligibility criteria. Eight studies were conducted within the past 5 years, with China as the most common study location. Only 2 studies investigated life participation as a primary study outcome. Eight studies targeted personal-physical barriers to life participation, 8 targeted multiple barriers, and 1 targeted an environmental-institutional barrier. Life participation was assessed within a subdomain of a broader quality of life assessment (The Kidney Disease Quality of Life [KDQOL]-36 or the 36-Item Short-Form Health Survey [SF-36]) in 11 studies. The majority of assessments captured life participation in all major domains of participation (self-care, work, and leisure).

**Limitations:**

Eligibility screening at title/abstract stage was not performed in duplicate; articles not available in English were excluded.

**Conclusions:**

Life participation has infrequently been prioritized as a health outcome in peritoneal dialysis (PD). Interventions have been narrow in focus given the range of challenges faced by people on PD and the holistic approaches used in other clinical populations. Future research should prioritize life participation as a key health outcome in PD and investigate the impact of interventions that address cognitive, affective, and environmental barriers to participation.

## Abrégé

**Contexte:**

Vivre avec l’insuffisance rénale peut entraver la participation à la vie (c.-à-d., la participation aux activités significatives du quotidien). La participation à la vie a récemment été identifiée comme un résultat de santé prioritaire pour les personnes sous dialyse péritonéale. Pourtant, elle demeure un sujet relativement inexploré en contexte de dialyse péritonéale (DP).

**Objectifs:**

Décrire les interventions utilisées pour promouvoir la participation à la vie dans une population sous dialyse péritonéale et mettre en évidence les lacunes de la recherche qui justifieraient une étude plus approfondie.

**Conception:**

Un examen de la portée a été effectué selon la méthodologie de l’Institut Joanna Briggs

**Sources:**

Consultation de six bases de données électroniques (MEDLINE [OVID], embase, PsycINFO, Cochrane Central Register of Controlled Trials, CINAHL plus et SCOPUS).

**Sujets:**

Des adultes (18 ans et plus) sous dialyse péritonéale.

**Mesures:**

Toute échelle ou sous-échelle dédiée mesurant la participation à la vie comme un résultat isolé.

**Méthodologie:**

La sélection du titre ou du résumé des articles a été effectuée de façon indépendante une fois la valeur adéquate de fiabilité inter-évaluateurs (kappa > 0,8) atteinte parmi les évaluateurs. L’examen du texte intégral et l’extraction des données ont été effectués en double. L’analyse des données extraites a été réalisée à l’aide de dénombrements, de pourcentages et de synthèses narratives afin de décrire les tendances dans la littérature.

**Résultats:**

Des 13 874 résultats répertoriés, seules 17 études répondaient aux critères d’admissibilité. Huit études avaient été menées dans les cinq dernières années, le plus souvent en Chine. Seules deux études avaient examiné la participation à la vie comme critère de jugement principal. Huit études ciblaient les obstacles personnels-physiques à la participation à la vie, huit ciblaient les obstacles multiples et une seule ciblait les obstacles environnementaux-institutionnels. La participation à la vie avait été évaluée dans un sous-domaine d’une évaluation plus large de la qualité de vie (KDQOL-36 ou SF-36) dans onze des études retenues. La majorité des évaluations portaient sur la participation à la vie dans tous les domaines principaux (soins personnels, travail et loisirs).

**Limites:**

La vérification de l’admissibilité à l’étape de la sélection des titres ou résumés n’a pas été effectuée en double; les articles non disponibles en anglais ont été exclus.

**Conclusion:**

La participation à la vie a rarement été considérée comme un résultat de santé prioritaire en DP. Les interventions ont été limitées, compte tenu de l’éventail des défis auxquels sont confrontées les personnes sous DP et des approches holistiques utilisées dans d’autres populations cliniques. Les recherches futures devraient accorder la priorité à la participation à la vie comme résultat clé de la santé des personnes sous DP et étudier l’effet des interventions qui s’attaquent aux obstacles cognitifs, affectifs et environnementaux qui entravent la participation à la vie.

## Introduction

In recent years, initiatives such as “Standardized Outcomes in Nephrology (SONG)” have aimed to ensure that health outcomes evaluated in research align with patient priorities (SONG).^1^ Through the SONG-Peritoneal Dialysis subproject, life participation has emerged as one of the top priorities of people on maintenance peritoneal dialysis (PD) therapy.^2^ Life participation refers to the ability to participate in valued life activities, including self-care activities (eg, bathing, dressing, cooking), work activities (eg, working, volunteering, managing the household), and leisure activities (eg, socializing, relaxing, playing games).^2,3^ Although peritoneal dialysis is often preferred to in-center hemodialysis because of the superior freedom and flexibility it affords patients,^4^ people on PD are still known to experience challenges with their life participation that affect their quality of life.^5-10^ In the global Peritoneal Dialysis Outcomes and Practice Patterns (PDOPPS) study, more than 50% adults on PD required assistance to complete routine self-care activities such as bathing, cooking, or dressing.^6^ The same study found that unemployment rates surpassed 50% among working-age adults on PD in most countries,^7^ whereas it is also common for people on PD to report difficulties engaging in social and leisure activities that they value.^10^ Participation in valued life activities is a crucial aspect of health and quality of life that provides people with a sense of purpose, autonomy, and identity,^11,12^ whereas disability is linked to poor outcomes including social isolation,^13^ poor mental health,^14^ increased care needs,^15^ and morbidity.^16,17^ It is therefore important to explore all possible pathways for improving life participation in people on PD therapy.

It is well-established in major rehabilitative models such as the International Classification of Function that life participation is the product of an interaction between personal, environmental, and task-related factors.^18^ Some models, such as the Canadian Model of Occupational Performance and Engagement, further break down these factors into personal factors that are physical, cognitive or affective, and environmental factors that relate to one’s physical, social, cultural, or institutional environment.^3^ A holistic approach to supporting life participation involves considering and addressing each of these factors in the treatment planning process,^3^ and this approach is widely recommended in best practice guidelines for people with various complex health conditions. For example, the Canada Best Practice Guidelines for Stroke Recovery suggests a range of multifaceted supports that include physical and cognitive rehabilitation, emotional support, caregiver education, and home modifications.^19^ A comprehensive approach to reablement in dementia similarly recommends diverse supports that target medical management, cognitive disability, physical function, acute injury or illness, assistive technology, supportive care, and caregiver support.^20^ Although there is limited evidence to elucidate the mechanisms of disability in peritoneal dialysis, available literature suggests people on PD similarly experience complex and varied disease-related sequelae that warrant a holistic approach to optimize their life participation. However, life participation is a relatively novel concept in the PD literature, and rehabilitation-based perspectives are traditionally underrepresented in the field of nephrology.^21-23^ As such, the nature of interventions that have been used to improve life participation in the PD population is currently unknown. The objective of this scoping review is therefore to identify and characterize intervention approaches that have been studied to promote life participation in people on chronic peritoneal dialysis, with a broader goal of identifying key research gaps and research priorities to advance knowledge in this field.

## Methods

### Research Design

A scoping review was conducted according to the guidelines of the Joanna Briggs Institute (JBI) methodology.^24^ Scoping review methodology was employed as it uses a rigorous, systematic approach to identify and analyze relevant literature pertaining to a broad research question or set of questions.^25^ For this review, scoping review methodology enabled us to describe patterns within a diverse body of literature on approaches to promoting life participation in people on PD. Articles and abstracts published up to March 2023 were considered for the review. Reporting was in accordance with the guidelines of the Preferred Reporting Items for Systematic Reviews and Meta-Analysis Extension for Scoping Reviews (PRISMA-ScR) checklist.^26^

### Inclusion Criteria

#### Types of participants

Eligible studies investigated adults aged 18 years and over with kidney failure who were undergoing chronic PD treatment. Studies focusing on children or youth under the age of 18 years, people receiving in-clinic dialysis and/or other types of dialysis (eg, hemodialysis), or those who underwent a kidney transplant were excluded, as their experiences of life participation and needs for interventions might differ from adults on PD.

#### Concept

Any study that investigated the impact of an intervention on life participation was included in the review. Life participation was defined as “the ability to participate in key activities of daily living including work, study, family, travel, hobbies, recreational and social activities.” Studies were eligible if they investigated participation in at least 1 meaningful life activity as an outcome; however, the studies that evaluated *components* or *determinants* of life participation (eg, walking speed, strength, cognitive functioning), but did not directly assess participation in 1 or more meaningful life activity, were excluded. Any study that reported life participation as a distinct and independent outcome (whether using a whole measure or a subscale of a measure) was eligible, whereas the studies that captured life participation as part of a broader concept (eg, quality of life) but did not isolate it as a distinct outcome were excluded.

#### Context

Studies from any geographic location or practice setting were eligible, with no limits on date of publication. Abstracts or full texts that were not available in English were excluded.

#### Types of sources

All primary research articles or abstracts (quantitative, qualitative, or mixed methods) were eligible for inclusion as long as they reported on the impact of an intervention on life participation in the PD population. Observational studies that assessed life participation, but did not examine it in relation to an intervention, were excluded. Case studies, case series, and opinion-driven reports were also excluded, owing to their non-use of scientific methodology.

### Data Collection

#### Search strategy and article selection

Six electronic databases were used as a part of the search strategy to identify eligible peer-reviewed articles: MEDLINE [OVID], EMBASE, PsycINFO, Cochrane Central Register of Controlled Trials, CINAHL Plus, and SCOPUS. Our search strategy began with a broader search population that included individuals with all stages of chronic kidney disease (CKD). Accordingly, search terms related to CKD, kidney failure, dialysis and/or kidney transplant were used in combination with search terms related to life participation and search terms related to interventions or treatments (Supplement 1). Titles and abstract screening was then conducted using Covidence software. First, 4 study reviewers participated in a pilot screening exercise for a subset of titles/abstracts, which was repeated until adequate inter-rater reliability was achieved (kappa > 0.8). Owing to the large number of results, title and abstracts were then divided and screened independently by the 4 reviewers. Articles selected for full-text review were subsequently categorized according to the following CKD subpopulations: pre-dialysis, hemodialysis, PD, and kidney transplant. For the purposes of this review, 2 reviewers conducted full-text screening of the PD articles in duplicate to identify eligible articles and consulted with a third reviewer to resolve disagreement about eligibility.

### Charting the Results

A data extraction table was developed a priori and piloted on 5 articles before full data extraction was undertaken. Two reviewers then performed independent data extraction in duplicate for each included study, as per the JBI methodology.^24^ Disagreements about data extraction were resolved by a third reviewer.

Data extracted included study authors, publication year, country, median sample size, and study design. Studies were noted as having investigated life participation as either the primary study outcome or a secondary outcome. Details about study interventions (name, description, provider) were extracted, as were the type(s) of life participation barriers they targeted according to the categories from the Canadian Model of Occupational Performance and Engagement^3^: Personal (physical, cognitive, or affective); Environmental (physical, social, cultural, institutional); or Occupational (Table 1). When there was a lack of clarity about what type of life participation barrier was being targeted, articles were re-read in full by 2 reviewers and the proposed mechanisms of the intervention were discussed until consensus was reached. Assessments used to measure life participation were documented, as were the specific life participation domains (self-care, work, or leisure) each study measured. Finally, the reported impacts of each intervention on life participation were categorized as “positive,” “mixed,” or “negative” to explore general trends in the literature. As this was a scoping review, no evaluation of evidence quality was conducted.

**Table 1.** Life Participation Barriers According to the Canadian Model of Occupational Performance and Engagement (CMOP-E).^3^

Life participation barrier type		Description	Examples that could affect PD population
Personal	*Physical*	Movement, strength, coordination, balance, endurance, pain, appearance, and physical illness and/or injury of body systems and/or structures	Progressive and generalized loss of muscle mass Physical symptoms (eg, fatigue, shortness of breath, pain) Physical deconditioning from sedentary behavior
	*Cognitive*	Memory, orientation, concentration, intellect, insight, judgment, general knowledge	Moderate to severe global cognitive impairment Memory and executive functioning deficits
	*Affective*	Emotions, mood, affect, volition, body image, coping skills, and reaction and adaptation to illness or injury	Mood disorders (eg, depression, anxiety) Illness intrusion, stress and burnout from kidney disease and treatment Uncertain views or expectations of the future Negative changes in body image from PD or health-related complications
Environmental	*Physical*	Built and natural aspects of the environment, from micro (eg, type of doorknobs) to macro (eg, how society is built to provide accessible space)	Suboptimal physical design of home, workplace or community environments to support disease-related needs and circumstances Physical home environment crowded by PD supply storage
	*Social*	People in the environment, our relationships with them, the networks we can leverage when requiring assistance, and the general availability of assistance to support living	Caregiver stress and burnout affecting support provided for participation Incomplete understanding of CKD and/or PD among family and social network, affecting interpersonal relationships and support to participate
	*Cultural*	Microlevel, such as person or family rituals, and macrolevel, such as expectations and attitudes of the broader culture	Cultural or religious beliefs might conflict with optimal health and/or life management recommendations Communication or relationship-building barriers with health care team
	*Institutional*	Governmental and organizational structures, policies and practices, as well as hidden or under-considered social determinants of health	Insufficient opportunity to personalize PD treatment to meet life needs or priorities Frequency of medical appointments and/or hospital visits Constraints in access to health care supports, services, and/or equipment
Occupational		Groups of activities and tasks of everyday life, named, organized, and given value and meaning by individuals and a culture	Time and energy required to manage kidney disease and PD treatment Increased complexity of daily activities while on PD (eg, dietary planning) Contraindication of certain activities due to risk of PD exit site infection

#### Presentation of results

Data were analyzed using counts and percentages to describe patterns in the literature and were summarized using narrative synthesis.

### Ethical Statement and Patient Consent

As this was a scoping review of published literature, ethics approval and patient consent were not required.

## Results

### Descriptive Characteristics

**Figure 1.**
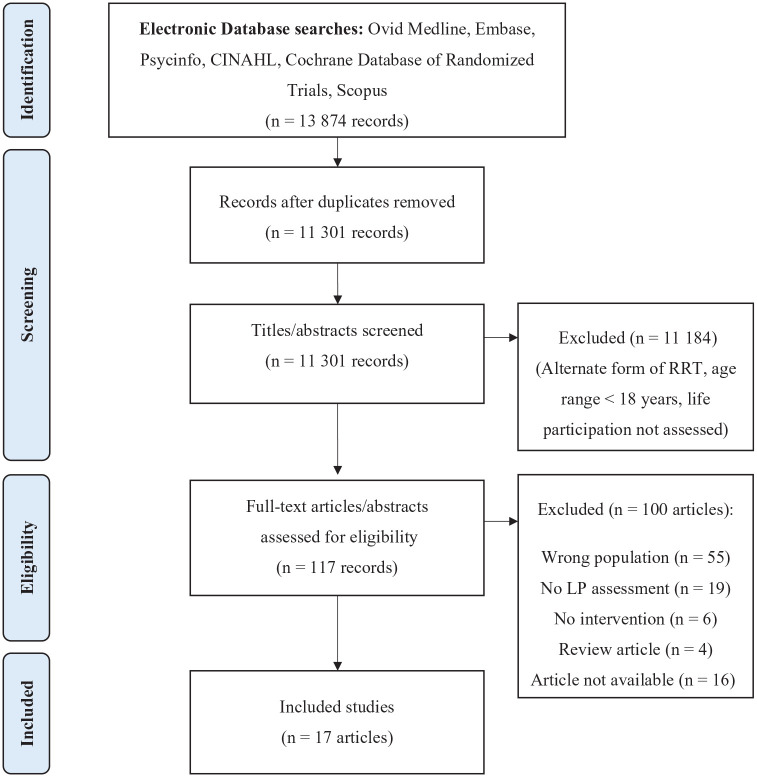
Scoping review PRISMA flow diagram.

We screened 13 874 studies, of which 17 met eligibility criteria and were found to be eligible for this review (Figure 1). The majority of studies (n = 8) were conducted between 2017 and 2022,^27-34^ with 3 studies conducted between 2012 and 2016^35-37^ and 6 studies conducted prior to 2011.^38-43^ The most common study location was China^29,32,33,35,38,40,42,43^; other locations included Canada,^31,41^ Italy,^28,34^ England,^36^ Japan,^27^ Sweden,^37^ Thailand,^30^ and the United States.^39^ The median study sample size was 36, and 9 studies (53%) were randomized controlled trials.^27-29,32,34-36,39,42^ Thirteen studies were published in medical journals,^27,28,31-36,38,39,41,43^ with the remainder of studies appearing in nursing journals^30,37,40,42^ or a nutrition journal.^29^ Study characteristics are outlined in Table 2.

**Table 2.** Evidence Chart.

Year	Title	First author	Country	Design	Sample size	Intervention type	Intervention description	Life participation focus	Life participation measure(s)	Reported results
2021	Clinical outcomes, quality of life, and costs evaluation of peritoneal dialysis management models in Shanghai Songjiang District: A multicenter and prospective cohort study	Xiaoyan Ma	China	Non-RCT	190	Institutional (environmental)	Family-community-hospital 3-level comprehensive management model with hospital-based preparation and training, community PD unit monitoring, and patient/family self-care	Secondary	Short Form-36 role-physical, role-emotional, social functioning subscales	Mixed
2021	Effects of probiotics on malnutrition and health-related quality of life in patients undergoing peritoneal dialysis: A randomized controlled trial	Yangbin Pan	China	RCT	116	Physical (personal)	A daily dose of a probiotic (13 109 CFU/d, ie, 2 capsules, tid) consisting of *Bifidobacterium longum, Lactobacillus bulgaricus*, and *Streptococcus thermophilus*	Secondary	Short Form-36 role-physical, role-emotional, social functioning subscales	Mixed
2021	Efficacy of physical exercise in peritoneal dialysis patients: A secondary analysis of the excite trial	Antonio Panuccio	Italy	RCT	35	Physical (personal)	Personalized home-based walking exercise program	Secondary	Kidney Disease Quality of Life Questionnaire role-physical, role-emotional, social functioning subscales	Mixed
2020	The effects of continuous home nursing visits on patients with chronic renal failure and peritoneal dialysis	Fei Li	China	RCT	72	Mixed (physical, affective)	Home nursing visits to address psychological and dietary needs as well as family education and support	Secondary	Quality of Life Questionnaire-C30 role function, social function subscales	Positive
2020	Effectiveness of a self-management retraining program improving the quality of life of people receiving continuous ambulatory peritoneal dialysis	Pungchompoo, Wanicha	Thailand	Non-RCT	41	Mixed (physical, cognitive, affective, social)	Self-management retraining program addressing disease education, self-efficacy, goal-setting, and psychoemotional support	Secondary	The Self-Management Behavior Questionnaire (SMBQ)	Positive
2020	A Proof-of-Concept Investigation of an Energy Management Education Program to Improve Fatigue and Life Participation in Adults on Chronic Dialysis	Farragher, Janine F	Canada	Non-RCT	5	Mixed (physical, occupational)	Energy management education and problem-solving training to promote life participation goal attainment	Primary	Canadian Occupational Performance Measure Performance and satisfaction subscales	Positive
2018	Home-based aerobic and resistance exercise training on peritoneal dialysis patients: A randomized controlled trial	Uchiyama, K	Japan	RCT	47	Physical (Personal)	Home-based aerobic exercise and resistance training program	Secondary	Short Form-36 role-physical, role-emotional, social functioning subscales	Positive
2017	Exercise in Patients on Dialysis: A Multicenter, Randomized Clinical Trial	Manfredini, Fabio	Italy	RCT	296	Physical (personal)	Personalized home-based walking program with gradually increased speed as program progressed	Secondary	Kidney Disease Quality of Life Questionnaire role-physical, role-emotional, social functioning subscales	Negative
2014	Long-term clinical effects of treatment by daytime ambulatory peritoneal dialysis with an individualized dialysis dose mode are comparable to traditional dialysis methods (hemodialysis or continuous ambulatory peritoneal dialysis) for end-stage renal failure	Zhi-yong, Zhang	China	RCT	32	Physical (personal)	Daytime ambulatory PD with an individualized dialysis dose	Secondary	Barthel Index; employment rates	Positive
2013	A randomized controlled trial to evaluate the effectiveness of a cognitive behavioural group approach to improve patient adherence to peritoneal dialysis fluid restrictions: A pilot study	Hare, Jennifer	England	RCT	15	Physical (personal)	Group-based liquid intake education and management program	Secondary	Short Form-36 role-physical, role-emotional, social functioning subscales	Positive
2012	Evaluation of an individual sleep intervention programme in people undergoing peritoneal dialysis treatment	Yngman-Uhlin, Pia	Sweden	Non-RCT	28	Mixed (physical environment, occupational)	Individual sleep hygiene and sleep scheduling intervention with a pressure-relieving mattress provided	Secondary	Short Form-36 role-physical, role-emotional, social functioning subscales	Mixed
2010	Multidisciplinary care program for patients on long-term peritoneal dialysis	Lam, M.F.	China	Non-RCT	36	Mixed (physical, affective)	Multidisciplinary care program provided by medical and allied health groups	Primary	Kidney Disease Quality of Life Questionnaire role-physical, role-emotional, social functioning subscales; Osteoporosis Assessment Questionnaire	Positive
2010	Health-related quality of life in patients undergoing peritoneal dialysis: effects of a nurse-led case management programme	Chow, Susan Ka Yee	China	RCT	100	Mixed (physical, affective)	Nurse-led case management program with a comprehensive pre-discharge assessment and individual education program prior to leaving the hospital, and a weekly telephone follow-up for 6 weeks after discharge	Secondary	Kidney Disease Quality of Life Questionnaire role-physical, role-emotional, social functioning subscales	Positive
2009	Promoting self-management improves the health status of patients having peritoneal dialysis	Su, Chun-Yan	China	Non-RCT	30	Mixed (cognitive, affective)	Self-management support program with various forms of education such as group discussion and individual consultation	Secondary	Karnofsky Performance Index	Positive
2005	The effect of a Tai Chi exercise program on quality of life in patients on peritoneal dialysis: A pilot study	Mustata, Stefan	Canada	Non-RCT	9	Mixed (physical, affective)	Tai Chi Wu-style–based exercise training program provided in the PD unit	Secondary	Short Form-36 role-physical, role-emotional, social functioning subscales	Positive
2002	Early quality of life benefits of icodextrin in peritoneal dialysis	Guo, Amy	The United States	RCT	93	Physical (personal)	Icodextrin dialysis solution administered over 13 weeks	Secondary	Short Form-36 role-physical, role-emotional, social functioning subscales	Positive
1998	Benefits of exercise training in patients on continuous ambulatory peritoneal dialysis	Lo, C Y	China	Non-RCT	20	Physical (personal)	Home-based exercise training program with dialysate inside abdomens (patients performed exercises on a treadmill, ski training machine, and upper limb and bike ergometers)	Secondary	KDQOL role-physical, role-emotional, social functioning subscales	Negative

### Intervention Types

The majority of life participation barriers targeted in PD interventions were categorized as personal-physical barriers (see Table 1 for description). Eight interventions (47%) used approaches such as exercise training to promote physical fitness^27,28,38^; an icodextrin dialysis solution to improve PD ultrafiltration^39^; daily probiotics to reduce malnutrition^29^; a liquid intake management program to prevent fluid overload^36^; and an individualized daytime ambulatory PD schedule to improve dialysis clearance.^35^ Eight interventions (47%) targeted more than 1 type of life participation barrier, using approaches such as self-management training to promote physical, cognitive, and emotional well-being^30,40^; a Tai Chi Wu-style exercise training program to enhance physical fitness, concentration and relaxation^41^; a sleep hygiene education and pressure-relieving mattress intervention to improve sleep^37^; an energy management intervention combined with problem-solving training to help patients improve their problem-solving and reduce fatigue^31^; and a regular home-based follow-up regimen post-PD initiation that targeted physical and emotional health through extra support and education to patients and families.^32^ One study targeted an institutional-environmental barrier to life participation, using a broadened 3-level family, community and hospital-based care model to better support PD patients with their disease management.^33^ No interventions exclusively targeted cognitive or affective barriers to life participation, nor did any studies exclusively address physical, social or cultural environmental barriers. Interventions are further described in Table 2.

### Life Participation Measures

Life participation was almost exclusively investigated as a secondary outcome in the identified studies,^27-30,32-42^ with only 2 studies focusing on it as a primary outcome.^31,43^ It was most frequently captured within broader quality of life assessments, with 11 studies (65%) exclusively relying on the role-physical, role-emotional, social functioning, and/or quality of social interaction subscales from the 36-Item Short-Form Health Survey SF-36 or the Kidney Disease Quality of Life (KDQOL)-36 assessments^27-29,33,34,36-39,41,42^ to assess life participation. Six studies (35%) employed other measures of life participation, which included the Canadian Occupational Performance Measure,^31^ QLQ-30 (Role Function and Social Function subscales),^32^ Karnofsky Performance Index,^40^ Barthel Index, Self-Management Behaviour Questionnaire (Role Management subscale), and rates of employment.^35^ Life participation measures were almost exclusively based on self-reported information, with only 1 study using an objective indicator (ie, employment status).^35^ The majority of studies (76%) captured all 3 major domains of life participation (self-care, work, and leisure),^27-29,31-34,38-43^ whereas 3 captured only self-care^30,36,37^ and 1 captured only self-care and work.^35^ Eleven studies reported exclusively positive life participation outcomes associated with their respective interventions,^27,30-32,35,36,39-43^ whereas 4 studies reported mixed results^28,29,33,37^ and 2 reported negative results^34,38^ (Table 2).

## Discussion

In this scoping review, we sought to identify and describe the literature on interventions that have been used to promote life participation in people on PD therapy. We found that life participation has been infrequently investigated as a priority health outcome in the PD literature, with only 2 intervention studies targeting it as a primary outcome. The barriers addressed to promote life participation were narrow in focus, with the majority of studies targeting personal-physical barriers such as physical fitness or dialysis adequacy and few studies targeting other potentially key barriers, such as cognitive impairments, mood disorders, and/or environmental barriers. We also found a lack of validated assessments used to capture changes in life participation, with the majority of outcome data being derived from subscales of broader health-related quality of life measures. Our findings demonstrate the need to use holistic models of disability to inform the life participation interventions employed for this population and to develop and validate measurement tools that can reliably capture change in this priority health outcome.

Our finding that interventions in the PD literature predominantly targeted personal-physical barriers to life participation is inconsistent with the range of disease-related sequelae and complications people on PD experience in their everyday lives. For example, visual impairments,^44^ cognitive dysfunction,^45^ and mood disorders^46^ are highly common in PD and are well-established risk factors for disability. People on PD are at risk of encountering physical, social, and/or institutional barriers to participation in their home, work, or community environments due to their disease-related challenges, eg, the physical space taken up by PD supplies in the home was identified as one of its major drawback in PDOPPS.^4^ They are undoubtedly also impacted by the exorbitant time and energy they must dedicate to managing kidney failure (eg, maintaining an intensive dialysis schedule, meeting complex dietary requirements, managing multiple medications). A holistic approach to promoting life participation that addresses its multifactorial determinants is therefore clearly needed for the PD population. There are already a plethora of promising rehabilitation-based approaches that could be applied to address key challenges experienced by this population. For example, cognitive rehabilitation interventions such as metacognitive problem-solving training and memory training are widely used in populations who experience executive functioning and memory impairments^47^ and could help to improve independence, participation, and treatment adherence in people on PD. Behavioral activation is an approach that uses activity scheduling and structured routines to address depression-related anhedonia^48^ and could be an excellent candidate for adaptation to the dialysis population to assist with dialysis-related time management and scheduling challenges. Environmental-based interventions, such as assistive equipment, home adaptations, caregiver education, and enhanced community services, are also important considerations for constructing a holistic approach to enabling life participation in PD. Future research should thus engage key PD and rehabilitation stakeholders to prioritize rehabilitative interventions to explore in the PD setting, and clarify design features that will enhance their feasibility and acceptability for this population.

Beyond the limited scope of interventions that have been used to enable life participation in PD, we generally found that life participation has rarely been studied as a dedicated trial outcome in PD. Although another recent review similarly reported on the amount of research activity on the topic of life participation in PD, our review uniquely excluded studies in which life participation was captured within a broader concept (eg, quality of life) but was not isolated from other concepts in the analysis and reporting of the outcome data. This tightened eligibility criteria allowed us to further evaluate the extent of research interest on this topic. Our finding that only 8 clinical trials and 17 intervention studies in PD have specifically assessed life participation as an outcome is striking and underscores the disparity that can develop between patient and researcher priorities when patients are not adequately engaged in the research process.^49^ Given that people on PD consider life participation to be one of the main goals of PD therapy and an indicator of treatment success that holistically encompasses what is important in their lives,^2^ greater efforts must be made to include life participation as a key trial outcome. The identification of life participation as one of the 6 core health outcomes in the SONG-PD initiative should help to address this knowledge gap over time, as life participation will be prioritized for inclusion in PD clinical trials moving forward. However, our finding that only 2 intervention studies have targeted life participation as a primary outcome in PD is equally concerning and noteworthy, as it suggests a lack of prioritization of this outcome and few interventions that have been specifically designed to target life participation. Beyond its importance to quality of life, supporting life participation in PD has a variety of potential societal benefits such as decreased caregiver burnout, health care spending, and long-term care admissions.^13-15^ There is therefore a clear need to dedicate further resources to prioritize life participation as a key indicator of treatment effectiveness in the PD population.

Although previous reviews sought to identify assessments for measuring life participation in PD, our review focused specifically on outcome measurement, in keeping with the notion that life participation should be a key target of change for interventions in PD. Our finding that life participation outcomes were predominantly measured using subscales from quality of life assessments is consistent with previous findings that dedicated measures of life participation are infrequently used in PD. In particular, the role-physical, role-emotional, and social functioning subscales from the SF-36 and KDQOL-36 assessments were the most common sources of intervention outcome information on life participation in the PD population. Although these subscales offer certain strengths (eg, they are brief and easy to complete, capture multiple domains of life participation, and assess changes in an individual’s self-perceived “normal” activities), they also have important limitations that have been noted in previous reviews, such as the limited reliability and validity data to support their use as stand-alone measures. We note that they also have questionable responsiveness to capture change for intervention research, eg, some only ask “yes/no” questions about life participation, leaving little room to assess change over time. The SONG-PD initiative to develop a PD-specific outcome measure of life participation for clinical trials will help to address some of these limitations, as it has been developed to maximize its validity^50^ and is undergoing psychometric testing to establish its reliability and responsiveness. However, as this outcome measure will be brief with limited descriptive detail, we support previous assertions that it should be supplemented with more comprehensive outcome measures of life participation in trials where life participation is a primary focus.^50^ There are a variety of such measures used in rehabilitation research that have data to support their responsiveness and patient-centeredness, and validating these measures in kidney disease populations should be a priority for future research.

Our review has a number of strengths. We followed the gold-standard JBI guidance^24^ on scoping review conduct to maximize its quality and thoroughness, and both the protocol and the final manuscript adhere to the PRISMA-SCr reporting guidelines.^26^ Our review used a comprehensive and systematic literature search, and we conducted duplicate full-text screening and data extraction of eligible articles. The limitations of this review include those inherent to scoping review methodology, such as a lack of critical appraisal of included articles. However, the objective of a scoping review is to lay the necessary groundwork for future systematic reviews by identifying research gaps and charting a course for knowledge advancement. Furthermore, due to the large number of initial search results we expect to find and resource limitations among our team, we were unable to perform full duplication of title and abstract screening. However, we conducted inter-rater validation for a subset of articles to ensure consistent screening prior to undertaking independent screening. Finally, we excluded non-English studies from the review due to resource limitations within our team, which may limit the generalizability of our findings to non-English populations.

## Conclusions

Life participation has been underemphasized as a priority health outcome in PD intervention research. The breadth of interventions explored to promote life participation in the PD population has been narrow in scope relative to the range of barriers faced by this population and the diverse intervention approaches used in other clinical populations with similar challenges. Future research should explore the potential for evidence-based interventions targeting cognitive, affective, and environmental barriers to enhance life participation in the PD population.

## Supplemental Material

sj-docx-1-cjk-10.1177_20543581241263168 – Supplemental material for Characterizing Interventions Used to Promote Life Participation in Adults on Peritoneal Dialysis Therapy: A Scoping Review Supplemental material, sj-docx-1-cjk-10.1177_20543581241263168 for Characterizing Interventions Used to Promote Life Participation in Adults on Peritoneal Dialysis Therapy: A Scoping Review by Alexia Kateb, Kaleigh McCarthy and Janine Farragher in Canadian Journal of Kidney Health and Disease
