# Supplementary material for: Characterizing Interventions Used to Promote Life Participation in Adults on Peritoneal Dialysis Therapy: A Scoping Review
Source: Can J Kidney Health Dis. 2024 Jul 30;11:20543581241263168. doi: 10.1177/20543581241263168 (PMC11292723; doi:10.1177/20543581241263168)
Supplement: sj-docx-1-cjk-10.1177_20543581241263168 – Supplemental material for Characterizing Interventions Used to Promote Life Participation in Adults on Peritoneal Dialysis Therapy: A Scoping Review [file sj-docx-1-cjk-10.1177_20543581241263168.docx]

**Supplement 1:** Review Search Terms

| **Population terms** | **Condition terms** | **Intervention terms** |
| --- | --- | --- |
| Chronic kidney disease Chronic kidney failure Chronic renal disease  Chronic renal failure  Chronic peritoneal dialysis  Chronic dialysis  End-stage renal disease End-stage renal failure End-stage kidney disease End-stage kidney failure Dialysis  Peritoneal dialysis  Renal  Kidney | Occupation  Occupational engagement  Occupational performance  Functional status  Quality of life  Participation  Life participation  Social participation  Meaningful activity  Activities of daily living  Work  Leisure  Self-care | Intervention program Treatment  Therapy  Rehabilitation  Remediation  Intervention approaches |
